# Antioxidant and Antiradical Activity of Coffee

**DOI:** 10.3390/antiox2040230

**Published:** 2013-10-15

**Authors:** Alexander Yashin, Yakov Yashin, Jing Yuan Wang, Boris Nemzer

**Affiliations:** 1 InterLab, Inc., Selskohozyaistvennaya 12a, Moscow 129226, Russia; E-Mails: yashinchrom@mail.ru (A.Y.); yashin@interlab.ru (Y.Y.); 2 VDF FutureCeuticals, Inc., 2692 N. State Rt. 1-17, Momence, IL 60954, USA; E-Mail: rwang@futureceuticals.com

**Keywords:** antioxidants, antioxidant activity, ORAC, amperometry, coffee

## Abstract

This review summarizes published information concerning the determination of antioxidant activity (AA) in coffee samples by various methods (ORAC, FRAP, TRAP, TEAC, *etc*.) *in vitro* and limited data of antiradical activity of coffee products *in vitro* and *in vivo*. Comparison is carried out of the AA of coffee Arabica and coffee Robusta roasted at different temperatures as well as by different roasting methods (microwave, convection, *etc*.). Data on the antiradical activity of coffee is provided. The antioxidant activity of coffee, tea, cocoa, and red wine is compared. At the end of this review, the total antioxidant content (TAC) of coffee samples from 21 coffee-producing countries as measured by an amperometric method is provided. The TAC of green and roasted coffee beans is also compared.

## 1. Introduction

Studies carried out in recent decades have confirmed that excessive accumulation of oxygen and nitrogen reaction products in body fluids including free radicals, such as the superoxide anion, hydroxyl radical, hydroperoxyl radical, *etc*., is a major cause of pathological changes in the human body, resulting in premature aging and numerous diseases.

The steady increase of free radicals in cells creates the conditions for so-called oxidative stress, wherein free radicals oxidize blood vessel walls, protein molecules, DNA, carbohydrates, and lipids. These radicals are particularly active in interacting with membrane lipids that contain unsaturated bonds, and thus alter the properties of cell membranes. The most active free radicals break bonds in DNA molecules and damage the cells’ genetic apparatus regulating their growth, which can result in cancerous cells. Oxidized low-density lipoproteins can be deposited on blood vessel walls which leads to atherosclerosis and cardiovascular disease [1,2,3].

Oxidative stress also plays a key role in the pathogenesis of aging [4,5]. Oxidative stress can be caused by various negative impacts, such as gamma or UV radiation, environmental factors, polluted and poor-quality food, stress, some medications or treatments, smoking, alcoholism, *etc*.

Prolonged oxidative stress inevitably leads to dangerous diseases—such as cancer, cardiovascular diseases, or diabetes—and premature aging. Oxidative stress can be reduced by antioxidant therapy, *i.e.*, by consumption of certain amounts of natural antioxidants contained in vegetables, fruits, berries, vegetable oils, honey, tea, coffee, cocoa, juices, wine, sprouted grains, and other foods [6,7,8]. However, in order to control consumption of antioxidants, it is necessary to know their content in foods and beverages. In this regard, quantitative measurement of antioxidants in foods and beverages and compilation of a corresponding databank becomes a highly important objective. Coffee is one of the major sources of antioxidants in people’s daily diet.

## 2. Review of Publications on Determination of Coffee Antioxidant Activity *in Vitro*

Beneficial health effects of coffee are usually attributed to its high antioxidant activity (ability to inhibit the process of oxidation). Many publications provide comparison of the antioxidant activity in such popular beverages as coffee, tea, and cocoa [9,10,11].

Antioxidant activity of coffee is related to chlorogenic, ferulic, caffeic, and *n*-coumaric acids contained in it [12]. In roasted coffee, melanoidins (brown pigments) are synthesized—these are strong antioxidants [13]. In some publications, caffeine and trigonelline are considered to be antioxidants also [14]. Phenylalanines which are formed during the roasting process show high antioxidant activity [14], as do heterocyclic compounds [15].

Many publications explore the relationship between coffee antioxidant activity and roasting, for example:

– Comparison of antioxidant activity in green coffee beans with roasted coffee [16,17];
– Dependence of antioxidant activity on roasting temperature and time [17];
– Relation between the antioxidant activity and roasting method used (convection and microwave roasting) [17].

Many researchers believe during the roasting process that Maillard reaction products, which are also strong antioxidants, are formed [12,16,17].

The antioxidant activity of volatile compounds, such as furans, pyrroles, formed by pyrolysis of coffee, has also been studied [14].

Interestingly, the compound, such as chlorogenic acid and polyphenols, which contributed to the antioxidant activity in coffee, is geographically related [18]. The coffee fruit was found to have more chlorogenic acids (CGA) in Arabica coffee fruit planted in Mexico and India compared to the coffee fruit grew in China.

In addition, evidence indicates that extraction procedures could affect the antioxidants contents in coffee fruit as well as the caffeine content [19]. It has been shown that the antioxidant activity was high in coffee fruit extract with low caffeine concentration in comparison with coffee fruit powder.

Apart from coffee, the antioxidant activity after roasting was also measured in wheat, nuts, and some other food products.

The antioxidant activity of green and roasted coffee is compared in several publications [20,21,22].

## 3. Methods of Measuring the Antioxidant Activity and Antioxidant Content in Coffee

Many methods based on new reagents, model systems and devices were suggested to use for determining the antioxidant activity, and many reviews were published on this subject [23,24,25,26,27].

The antioxidant activity (AA) is measured by various chemical and physicochemical methods. All those methods are most often based on the direct or indirect measurement of reaction rate and/or completeness.

Three types of methods could be distinguished based on the following measurements:

– Oxygen intake;
– Formation of oxidation products;
– Uptake or binding of free radicals.

In the first and second cases, AA is determined based on the degree of inhibition or intake rate for reagents or the products formed.

Primary methods for the AA measurements are: ORAC—oxygen radical absorbance capacity; TRAP—total radical trapping antioxidant parameter; FRAP—ferric reducing antioxidant power; TEAC (Randox)—trolox equivalent antioxidant capacity; ABTS—2,2-azinobis(3-ethylbenzthiazoline)-6-sulfonic acid; TBARS—thiobarbituric acid reactive substance.

In all of these methods, the AA depends on multiple parameters, including time, temperature, nature of the substance, concentration of the antioxidants and other compounds, *etc*.

The antioxidant activity cannot be measured directly—what is typically measured is the effect of the antioxidants on the degree of oxidation. All of these methods often give conflicting results.

The drawback of many methods used for measuring antioxidant activity is the lack of proper substrates during the measurement process. Antioxidant activity is most often measured based on long-living synthetic free radicals (ABTS, DPPH, AAPH, *etc*.). Lots of synonymous terms have been proposed, including “antioxidant ability”, “antioxidant power”, “antioxidant activity”, and “antioxidant capacity” [28]. All of these terms are related to the antioxidant concentration (the activity of substances or substance groups).

Many well-known methods, such as TEAC, TRAP, FRAP, *etc*., are based on reduction reactions of long-living free radicals or Fe (III) complex.

Obviously, all these methods have drawbacks because they use synthetic free radicals which have nothing in common with free radicals in the human body.

The above methods are time-consuming and include several stages; the signals are recorded using expensive devices, such as spectrophotometric, fluorimetric, and chemiluminescent detectors.

Electrochemical methods are more promising for the measurement of integral antioxidant capacity because the reaction between active oxygen compounds in aqueous media is accompanied by electron transfer, *i.e.*, they are electrochemical in nature.

In [29] has been proposed to measure antioxidant capacity by using electrogenerated oxidizers: chlorine, bromine, and iodine. The electrogeneration of halogens during coulometric titration was performed at a constant current of 5.0 mA from 0.2 M aqueous solutions of KCl and KBr in 0.1 M H_2_SO_4_; titration end-point was detected by amperometric indication using two polarized platinum electrodes. The authors of this method were the first to introduce a characteristic called “bromine-scavenging antioxidant capacity” which is expressed in electricity units (coulombs) used for the titration of 100 g (or 100 mL) of the tested material by electrogenerated bromine.

It was demonstrated that the value of bromine-scavenging antioxidant capacity reflects the total content of antioxidants in foods, beverages, and extracts of medicinal plants. It should be noted, however, that bromine and iodine can oxidize not only the antioxidants but also other compounds.

In one publication, it has been proposed to measure antioxidant activity by a potentiometric method using a mediator solution of K_3_[Fe(CN)_6_]/K_4_[Fe(CN)_6_] [28].

This method was successfully used for the determination of the antioxidant activity of pure chemical compounds as well foods, beverages, dietary supplements, and extracts of medicinal plants.

Another electrochemical method proposed is cathodic stripping voltammetry. In this method, oxygen electroreduction is used as a model reaction.

In order to determine antioxidants in the tested substances, the dependences of the first oxygen reduction peak current on their concentration in the solution and the duration of the process.

In this method, a mercury film electrode (banned in many countries) was used as an indicator.

Antioxidant activity of many foods and biological samples have been measured by this method.

### 3.1. ORAC (Oxygen Radical Absorbance Capacity) Method

The ORAC, one of the methods most often used for the determination of antioxidant activity, especially in the U.S., was proposed by Cao G. [30] and later has been automated and validated by Prior, R., Ou, B. and other four different assays [31,32,33,34,35].

This method is based on measuring changes in fluorescence intensity *vs*. reaction time. Quantitative measurement of the antioxidant activity is carried out by determining the area between two curves which represent reaction with and without the antioxidant.

In the early tests, B-phycoerythrin was used as the fluorescent probe, which tended to show slightly lower results because this protein reacted with polyphenols. That is why it was later suggested to rather use fluorescein, a more stable fluorescent compound.

In this method, 2,2′-azobis-(2-amidinopropane) dihydrochloride (AAPH) is used as the source of peroxyl radicals. In the ORAC method, trolox, a water-soluble analog of vitamin E, is used as the standard. Fluorescence intensity is measured at 515 nm with the excitation wavelength of 485 nm.

Changes in the fluorescence intensity are registered every minute over 35 min, both with and without an antioxidant. Therefore, the total measurement lasts for 70 min. The results obtained for each sample are then calculated using calibration curves and presented in mmol/g of dry matter.

The overall mechanism of the ORAC method is as follows: In the solution, the AAPH reagent decomposes at 37 °C with the release of peroxide free radical. When an antioxidant, or a mixture of antioxidants, are added, fluorescence quenching decreases due to the fact that antioxidants neutralize the effects of free radicals on the fluorescein. The convergence of the method is in the form of standard deviation (SD) ± 15%.

In a 2005, Prior, *et al*. [36] wrote that a standardized total antioxidant capacity method should evaluate “effectiveness against various ROS/RNS such as superoxide anion, hydroxyl, and peroxynitrite...and this may require additional methods specific for each radical source”.

The new generation of ORAC assay–total ORAC for food and nutrition (Total ORAC FN) has been introduced by Brunswick Laboratories (Norton, MN, USA) in 2008. Total ORAC FN represents a breakthrough in comprehensive antioxidant testing for food and nutrition products as antioxidant activity against five of the most important free radicals found in human–hydroxyl (HORAC assay), peroxyl (ORAC assay), peroxynitrite (NORAC assay), singlet oxygen (SOAC assay), and superoxide anion (SORAC assay).

The total antioxidant capacity of whole coffee fruits Arabica and their extracts commercially produced by FutureCeuticals, Inc. (Momence, IL, USA) has been analyzed using Total ORAC FN assays [19]. The results of ORAC FN are shown in Table 1.

**Table 1:** Total ORAC activity coffee fruit sample [19]. (Note: ORAC FN results are expressed as µmole Trolox Equivalent (TE)/g ± SD.

Coffee Fruit Sample	Coffee Fruits Extract-1	Coffee Fruits Extract-2	Coffee Fruits Powder-1	Coffee Fruits Powder-2
Extration/Drying Procedure	Multistep ethanol extraction	Single step extraction	Freeze-dried	Air-dried
Total CGA	76.5%	45.0%	8.79%	4.53%
ORAC	15,246 ± 453	6097 ± 225	823 ± 86	735 ± 47
HORAC	41,389 ± 3447	18,709 ± 426	3520 ± 287	2140 ± 125
NORAC	1317 ± 104	527 ± 52	75 ± 11	52 ± 7
SORAC	2193 ± 1591	860 ± 24	271 ± 14	123 ± 62
SOAC	3422 ± 355	2042 ± 185	311 ± 13	239 ± 12
Total ORAC FN	64,354 ± 2584	28,237 ± 782	4768 ± 285	3439 ± 134

Table 1 shows that the total chlorogenic acid (CGA) contents correlates to total antioxidant capacity. The coffee extract with the highest chlorogenic acid contents corresponds to the highest total ORAC activity compared with whole coffee fruit powder (air dried and freeze dried) [19].

### 3.2. Amperometric Method for Determining Total Antioxidant Content

Amperometric method (AM) is based on measuring an electric current in the detector cell which occurs during oxidation of the analyte on the working electrode surface when certain potentials are applied. Thus, when an amperometric method is used, the changes in the current passing through the cell are registered—these changes directly correlate to the changes in the analyte concentration [1].

The amperometric method can operate in three modes: at a constant potential, at pulse potentials, and by scanning the potentials in the entire range.

The amount of electric current depends on the nature of the analyte, nature of the working electrode, and the potential applied to the electrode.

Emerging electric currents are very small, within 10^−6^ to 10^−10^ A. These analog signals are amplified, converted into digital signal by an analog-digital converter (ADC), and then displayed on a computer screen. If necessary, the outputs can be printed.

The working electrode is a glassy carbon electrode which is most commonly used in the determination of polyphenolic compounds. Ionization potentials of phenolic compounds vary within 100–1300 mV.

The signal is recorded as differential output curves. Using special software, the areas or peak heights (of the differential curves) are calculated for the analyte and for the reference substance. Well-known antioxidants, such as quercetin, dihydroquercetin, mexidol, trolox, gallic acid, *etc.* can be used as reference substances. The amperometric method has several advantages in the determination of antioxidant activity: not taking into account sample preparation, one determination takes only a few min; analysis (data recording and processing) takes place in real time; accuracy and reproducibility of the analysis is ensured by accurate dosing with a six-way valve; standard deviation (SD) of valve dispenses is less than 0.5%; SD of the successive measurements of the analyzed samples is less than 5%; limit of detection for polyphenols and flavonoids is at the level of nanograms and picograms (10^−9^–10^−12^ g). At such low concentrations, the likelihood of the mutual influence of different jointly present antioxidants, such as by a manifestation of synergy, is significantly reduced.

At the same time, high selectivity in determination only of antioxidants (compounds capable of inhibiting oxidation) is achieved; other compounds present in complex mixtures do not interfere with the determination of the antioxidants. No chemicals (other than standards) are required for this analysis; therefore, the cost of measurements is very low.

The amperometric method is the only method which allows for direct measurement of all antioxidants in a sample. The amperometric method has been successfully applied to determine the antioxidant capacity of various wines and coffee [37]. It was noted that this method is direct, accurate, objective, and fast. In one publication, this method was used to determine the antioxidant capacity of olive oil, *i.e.*, fat-soluble samples, from different Mediterranean countries—Italy, Greece, France, Spain, Morocco, Tunisia [38]. This method allows for the assessment of quality and authenticity of olive oil. In one study, the antioxidant power of lipophilic compounds present in vegetables, such as carotenoids, chlorophyll, tocopherols and capsaicin, was determined [39]. When applied to pure compounds, the order of antioxidant power resulted as follows (in descending order): lycopene > beta-carotene > zeaxanthin > alpha-carotene > beta-cryptoxanthin > lutein > α-tocopherol > capsaicin > chlorophyll a > chlorophyll b > astaxanthin > canthaxanthin. The results of the antioxidant activity measurements obtained for five vegetable and two fruit extracts were compared to those obtained by the ABTS. A good correlation between the two methods was found, except for spinach. The authors conclude that the amperometric method can be successfully used for direct, quick, and reliable monitoring of antioxidant power of lipophilic food extracts. Amperometric (electrochemical) method is also used to determine the antioxidant status in people.

## 4. Results of Antioxidant and Antiradical Activity of Coffee

### 4.1. Antioxidants and Antioxidant Activity

The most antioxidant-rich beverages are [1]: coffee—200–550 mg/cup; tea—150–400 mg/cup; red wine—150–400 mg/glass. Intake of these drinks makes a significant contribution to the total amount of antioxidants consumed by people.

Green coffee beans contain large amounts of polyphenolic antioxidants, such as chlorogenic, caffeic, ferulic, and *n*-coumarinic acids. Coffee roasting significantly alters the composition of polyphenols due to Maillard reaction (chemical reaction between amino acids and sugars).

A cup of coffee which contains 10 g of roasted coffee beans may have 15 to 325 mg of chlorogenic acids. On average in America, one cup contains approximately 200 mg of chlorogenic acid. Antioxidant activity of ferulic and caffeic acids was studied both *in vitro* and *in vivo*. Ferulic acid presented in coffee has anti-inflammatory, anti-allergic, antibacterial, antiplatelet, and antiviral effect [34]. Pharmacological properties of ferulic acid are related to its high antioxidant activity, in particular, its ability to inhibit lipid peroxidation in biological membranes.

In one study, it has been shown that ferulic acid at a concentration of 10^−3^ mol/L in a perfusion solution reduces arrhythmia [40].

People consume coffee or tea every day, sometimes several times a day. Coffee is the main drink in Europe, America and Asia. In recent years, consumption of coffee has increased even in England, a prominently tea-drinking country. Coffee consumption is rising rapidly in China and Japan where a centuries-old tradition of tea consumption is strongest.

Coffee and tea provide a very significant portion of the daily intake of antioxidants established for humans.

We have compared coffee, tea and cocoa in terms of the total antioxidant content (TAC). The results of the comparison are shown in the Table 2.

**Table 2:** Total antioxidant content of different beverages. Note: Natural roasted coffee produced by the Madeo Company. Best grades of green and black tea purchased in the stores. (Averaged values of TAC) [41].

Beverages	One-time consumption rate, in grams	TAC, mg/g
Coffee	7–10	150–300
Green Tea	2	150–300
Black Tea	2	110–200
Cocoa	10	200–250

The data presented in Table 2 suggest that in terms of antioxidant capacity (as total antioxidant content), coffee and cocoa are comparable with tea, which can be attributed to higher consumption rates of these products. The antioxidant capacity of tea is largely related to the catechins and their polymers (thearubigins, *etc.*).

Cocoa is the most balanced product in terms of antioxidants since it contains water-soluble and lipid-soluble antioxidants. This point towards an interesting combination of coffee and cocoa and some manufactures are attempting to create such mixtures.

Table 3 summarizes data obtained in Italy on the antioxidant activity of various non-alcoholic and alcoholic beverages [42]. It should be noted that figures provided for green and black tea as well as for beer are understated. According to our data, the antioxidant activity of cognac is higher than that of whisky.

**Table 3:** Comparison between the antioxidant activity (AA) of coffee and other drinks [42] (Notes: All samples were collected in Italy; FRAP—ferric reducing antioxidant power; TRAP—total radical-trapping antioxidant parameter; TEAC—trolox equivalent antioxidant capacity).

No.	Beverages	AA determined by different methods		
		FRAP (mol Fe^2+^/L)	TRAP (mol Trolox/L)	TEAC (mol Trolox/L)
1.	Coffee (Espresso)	129.4	66	36.5
2.	Coffee (Instant)	108.6	52.4	32.5
3.	Coffee (Extract)	96.4	59.6	30.3
4.	Coffee (Espresso, Decaffeinated)	93.0	45.8	27.0
5.	Red wine (Chianti)	31.5	14.8	11.4
6.	Green Tea	18.0	7.6	6.0
7.	Black Tea	10.1	4.9	3.6
8.	Rose wine (Villa Tofre)	8.3	2.2	2.4
9.	White wine (Pinot)	3.7	2.1	1.7
10.	Whisky	3.4	2.3	1.7
11.	Cognac	2.2	1.5	1.3
12.	Beer	2.8	–	1.0

Data of caffeine, theobromine, and total polyphenols content in standard cups of coffee and tea, as well as their antioxidant activity, are provided in Table 4 [20].

**Table 4:** Caffeine, theobromine and total polyphenols content in one cup of coffee and tea [20]. (Notes: Caffeine and theobromine were determined using high performance liquid chromatography (HPLC); the total content of polyphenols was determined by Folin-Ciocalteu assay).

No.	Description	Coffee	Tea
1.	Caffeine, mg/cup	181 ± 10	130 ± 7
2.	Theobromine, mg/cup	28.9 ± 1.1	5.9 ± 0.4
3.	The total content of polyphenols, gallic acid equivalents (GAE) mg/cup	161 ± 9	87 ± 9
4.	AA, Crocin test, mM TX equivalent/cup	7.4 ± 0.3	1.4 ± 0.1
5.	TRAP, mm ROO° equivalent/cup	10.1 ± 0.6	1.3 ± 0.1

Table 5 shows the effect of the roasting degree of Arabica and Robusta coffee beans on their antioxidant activity [11]. The antioxidant activity of the beverage is represented by the increase of the lag time (lag time of LDL in the presence of coffee beverage with respect to the lag time of control LDL). Results are expressed as mean (of three to five individual experiments) ± SD.

**Table 5:** Antioxidant activity of green and roasted Arabica and Robusta coffee beans [11].

No.	Type of Coffee	Increase of lag time, min	
		Arabica	Robusta
1.	Green	366 ± 74	643 ± 68
	Roasting degree:		
2.	−light	284 ± 80	294 ± 41
3.	−medium	206 ± 30	190 ± 39
4.	−dark	168 ± 23	134 ± 34

As can be seen from Table 5, the antioxidant activity of Robusta green coffee is significantly higher than that of Arabica. However, this difference virtually disappears after light roasting; and after dark roasting Arabica coffee even exceeds Robusta coffee with regards to antioxidant activity.

In one article, antioxidant activity of coffee, tea, and cocoa—the most widely consumed beverages containing polyphenolic antioxidants–was compared [11]. The beverages were prepared as 0.7%–2.5% soluble coffee and 1.5%–3.5% cocoa; teas (green, black, or herbal) were prepared as one tea bag brewed in 220 mL of hot water. These drinks are often taken with milk. It has been shown that addition of milk did not alter the antioxidant activity.

The currently used roasting methods—convection and microwave roasting—were compared in [17].

Data on the antioxidant activity measured by spectrophotometric (UV-vis) and HPLC methods, as well as data on caffeine content in various types of coffee and in coffee roasted by different methods, is presented in Table 6.

**Table 6:** Total polyphenols and caffeine content of raw and different roasted coffee beans [17].

No.	Type of coffee and roasting method	Total amount of polyphenols, %		Caffeine, %
		UV	HPLC	
1.	Green	7.88	7.15	2.12
	Roasted:			
2.	−convection	2.26	2.07	1.82
3.	−microwave	2.31	2.19	1.90
4.	−wet, convection	2.58	2.36	1.89
5.	−wet, microwave	2.75	2.64	2.07
6.	−dry, convection	2.06	1.97	1.86
7.	−dry, microwave	2.11	2.03	2.08
8.	−convectio microwave	3.03	2.87	1.95

As can be seen from Table 6, microwave roasting preserves antioxidants better than convection roasting, and the wet method for separating the beans from the fruits is preferable to the dry method by this criterion. The latter is probably related to the greater oxidation of the antioxidants in the dry method.

In one study, efficiency of extraction of biologically active compounds from the coffee powder was researched [43]. Extraction was carried out with ethyl acetate, ethyl ether, and dichloromethane. Ethyl acetate in acidic medium was shown to be the most effective.

The yellow-brown extract of Robusta coffee was separated by gel-filtration chromatography according to the molecular weight. Individual fractions were collected at the column outlet for further study of their antioxidant activity (using a β-carotene-lipoic acid model system). Protective activity (PA) against lipid oxidation was determined *in vitro*. The results of these studies are summarized in Table 6.

As can be seen from Table 7, only high-molecular weight fractions showed antioxidant activity and only low-molecular weight fractions had protective activity. These interesting data require further careful research.

**Table 7:** Dry residue, molecular masses, antioxidant activity (AA%), and protective activity (PA%) of coffee fractions obtained by gel filtration chromatography (GFC) of acidic extract [43].

No.	Sample	mg/mL ^a^	Molecular weight	AA, %	PA, %
1.	acidic coffee extract	1.31	–	96	100
2.	1st fraction	0.50	<6000	74	0
3.	2nd fraction	0.16	<550	37	0
4.	3rd fraction	0.13	350–550	0	0
5.	4th fraction	0.23	200–350	0	53
6.	5th fraction	0.29	<200	0	85

Note: ^a^ mg of dry residue per mL of brewed coffee.

In that same study, the pH of the coffee solutions (green and varying degrees of roasting) was also determined. Robusta coffee varieties from Zaire and Ecuador and Arabica coffee varieties from Brazil, Colombia, and Costa Rica were studied. According to the results of this research, all varieties of green coffee have a higher pH than roasted coffees. Values ranges from 5.77 to 5.95. For roasted Arabica coffee varieties the pH values were lower than for Robusta varieties: 5.01–5.39 and 5.43–5.68, respectively. As for the antioxidant activity, it is higher in green coffee than in roasted; at the same time, for all coffee varieties its value increased by 4%–6% with the increase of the roasting time (from 10 to 30 min).

### 4.2. Antiradical Activity

It should be emphasized that there is a difference between “antiradical” and “antioxidant” activity. The antiradical activity characterizes the ability of components to react with free radicals (in a single free radicals reaction), but antioxidant activity represents the ability to inhibit the process of oxidation (which usually involves a set of different reactions) [44].

In [45] have been shown that solutions of green and roasted coffee have significant antiradical activity against hydroxyl free radicals. This effect was studied both *in vitro* (using deoxyribose assay) and *in vivo* in biological cellular systems (IMR32 cells).

All solutions tested *in vitro* using the deoxyribose degradation assay have shown similar inhibitory activity. In the cell cultures, solutions of Robusta roasted coffee have shown greater activity than other solutions. Using preparative gel-filtration chromatography, the components of the roasted Robusta coffee were separated into three fractions by their molecular weight. The most active fraction was found to be the one containing the 5-*O*-caffeoylquinic acid. Activity of the solution increased when concentration of this acid increased (from 0.02 to 0.10 mM).

When roasted, the content of chlorogenic acids decreases but at the same time, the content of high molecular compounds, and melanoidins (also possessing antiradical activity in coffee) increases.

In order to confirm that the 5-*O*-caffeoylquinic acid has the greatest efficiency, solutions of coffee biologically active compounds were prepared and tested for their antiradical activity (see Table 8).

**Table 8:** Antiradical activity *in vitro* and *in vivo* of standard solutions of coffee biologically active components [45].

No.	Compounds	%	
		*in vitro*	*in vivo*
1.	5-*O*-caffeoylquinic acid	34.6 ± 7.5	264.3 ± 56.2
2.	Caffeine	27.2 ± 6.4	25.2 ± 5.2
3.	Nicotinic acid	19.2 ± 6.7	23.2 ± 4.8
4.	Trigonelline hydrochloride	19.3 ± 12.6	15.7 ± 4.1

Table 8 shows that 5-*O*-caffeoylquinic acid has the highest activity both *in vitro* and *in vivo*, particularly, *in vivo*. These results could explain the significant neuroprotective effects found for coffee consumption in recent epidemiological studies. These studies have established a direct link between coffee consumption and reduced risk of neurodegenerative diseases, including Parkinson’s disease [46,47,48].

Coffee and coffee beverages can inhibit *in vitro* the mutagenicity of oxidants, such as tert-butyl hydroperoxide, and therefore can inhibit lipid peroxidation and formation of malondialdehyde, a marker of oxidative stress [49].

### 4.3. Total Antioxidant Content by Amperometric Method

The amperometric measurements of total antioxidant content has been provided by amperometric detector or analyzer TsvetYauza-01-AA (Khimavtomatika, Moscow, Russia).

The device operates on specially selected modes whereby only antioxidants are determined, and other compounds do not interfere with their determination.

Samples to be used for the determination of the total content of antioxidants in coffee were prepared as follows: the roasted coffee beans were ground into a powder, then boiling water was added to the powder, and the mixture was brewed for 5–10 min. A sample was taken using a syringe and introduced to a six-way valve on the device. When the valve was turned from the “dosing” position to the “analysis” position, the mobile phase (eluent) captured the sample and transferred it to the cell of the amperometric detector.

The total antioxidant content (TAC) in roasted coffee beans (provided by coffee manufacturer Freshly Roasted Company; coffee varieties of the MADEO trademark) from major coffee producers in 21 countries, is shown in Table 9. Quercetin was used as a reference substance.

As can be seen from Table 9, the content of antioxidants in different varieties of coffee differs 1.5 times at most and depends on the country of origin, degree of roasting, coffee processing method, altitude at which the coffee was grown, the soil, the average temperature, and number of sunny days per year.

Drinking two or three cups of coffee made with roasted beans ensures the daily consumption rate for antioxidants. Unfortunately, only 20% of consumers use natural roasted coffee in Russia, whereas, for example, in the U.S., this figure is 80%. In recent decades, instant coffee grades have become widely used. True lovers of coffee, “coffee nerds”, undoubtedly prefer natural roasted coffee because this type of coffee has a unique taste and aroma.

**Table 9:** The total content of antioxidants (TAC) in MADEO coffee beans provided by company “Freshly Roasted Coffee” (quercetin used as standard) [41,50].

No.	Name	TAC, mg/g	TAC, mg/7 g
			(per cup)
1	Maragogype, Guatemala	32.1	224.7
2	Brazil	30.8	215.6
3	Colombia	30.5	213.5
4	Yirgacheffe, Ethiopia	28.6	200.2
5	Puerto Rico	28.6	200.2
6	Decaffeinated coffee	27.3	191.1
7	Kenya	27.0	189.0
8	Cuba	26.7	186.9
9	Jamaica	26.1	182.7
10	Sumatra	24.5	171.5
11	Mexico	24.1	168.7
12	Nicaragua	24.1	168.7
13	Guatemala	24.0	168.0
14	Honduras	23.9	167.3
15	Maragogype, Nicaragua	23.2	162.4
16	Maragogype, Mexico	23.1	161.7
17	Tanzania	23.1	161.7
18	Yemen	22.9	160.3
19	Costa Rica	22.5	157.5
20	Skybury, Australia	22.3	156.1
21	Java	21.1	147.7

Due to some concentration during the extraction procedure, some varieties of instant coffee may contain greater amounts of antioxidants than roasted coffee.

The TAC values for green and roasted coffee beans from several different countries in Africa, South America, and Asia are provided in Table 10. As can be seen from Table 9, Arabica roasted coffee beans showed higher TAC than green coffee beans, which is quite an unexpected result. Previously, similar results were observed in some studies which used other methods.

**Table 10:** Impact of coffee roasting process on the total antioxidant content (quercetin used as standard) [41,50].

No.	Type of Coffee	Producing Country	TAC, mg/g	
			Green	Roasted
1.	Arabica	Tanzania	34	43
2.	Arabica	Ethiopia (Yirgacheffe)	32	40
3.	Arabica	Brazil	30	41
4.	Robusta	Bali	65	63
5.	Robusta	Uganda	67	55

## 5. Conclusions

In the publications reviewed, the values for both antioxidant activity (AA) and total antioxidant content (TAC) of roasted coffee are at the same level as corresponding values for tea, cocoa, and red wine.

Roasting has a contradictory effect on TAC and AA. The HPLC results show that the content of chlorogenic acids, the main antioxidants in green coffee beans, significantly decreases after roasting. On the other hand, in the process of roasting such polymeric compounds as melanoidins (which are potent antioxidants) and other compounds are formed.
